# ECM‐Inspired Hydrogels with ADSCs Encapsulation for Rheumatoid Arthritis Treatment

**DOI:** 10.1002/advs.202206253

**Published:** 2023-01-22

**Authors:** Haofang Zhu, Xiangyi Wu, Rui Liu, Yuanjin Zhao, Lingyun Sun

**Affiliations:** ^1^ Department of Rheumatology and Immunology Institute of Translational Medicine The Affiliated Drum Tower Hospital of Nanjing University Medical School 321 Zhongshan Road Nanjing 210008 P. R. China; ^2^ Department of Rheumatology and Immunology The First Affiliated Hospital of Anhui Medical University 218 Jixi Road Hefei 230022 P. R. China; ^3^ State Key Laboratory of Bioelectronics School of Biological Science and Medical Engineering Southeast University 2 Sipailou Nanjing 210096 P. R. China

**Keywords:** bioinspired, encapsulation, hydrogel, peptide dendrimer, rheumatoid arthritis, stem cells

## Abstract

Due to their intrinsic anti‐inflammatory and immunomodulatory properties, adipose‐derived stem cells (ADSCs) are explored as a promising alternative in treating rheumatoid arthritis (RA). To address the poor survival and function loss of directly injected stem cells, efforts in this area are focus on the generation of efficient cell delivery vehicles. Herein, a novel extracellular matrix (ECM)‐inspired injectable hydrogel for ADSCs encapsulation and RA treatment is proposed. The hydrogel with dendritic polylysine and polysaccharide components is formed through the reversible Schiff base crosslinking. It possesses self‐healing capability, superior mechanical properties, minimal toxicity, and immunomodulatory ability. When encapsulated with ADSCs, the hydrogel could recover chronic inflammation by directly reversing the dominant macrophage phenotype from M1 to M2 and inhibiting the migration of fibroblast‐like synoviocytes. Through a collagen‐induced arthritis rat model, the tremendous therapeutic outcomes of this ADSCs‐laden hydrogel, including inflammation attenuation, cartilage protection, and bone mineral density promotion are demonstrated. These results make the ECM‐inspired hydrogel laden with ADSCs an ideal candidate for treating RA and other autoimmune disorders.

## Graphical abstract

A novel extracellular matrix (ECM)‐inspired hydrogel for adipose‐derived stem cells (ADSCs) encapsulation and rheumatoid arthritis (RA) treatment is developed. The hydrogel demonstrates self‐healing capability, superior mechanical properties, minimal toxicity, and immunomodulatory ability. In a collegan‐induced arthritis model, the ADSCs‐laden hydrogel displays great therapeutic outcomes, indicating its great potential for treating RA.

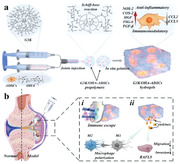

## 1 Introduction

Rheumatoid arthritis (RA), a chronic inflammatory disorder, is characterized by synovial hyperproliferation, macrophage infiltration, and dysregulated autoimmune responses.^[^ ^1^, ^2^, ^3^ ^]^ Adipose‐derived stem cells (ADSCs) are multipotent cells that can exert regulatory effects on various immunocytes.^[^ ^4^, ^5^, ^6^, ^7^ ^]^ It has been demonstrated that ADSCs are attractive candidates for RA treatment.^[^ ^8^, ^9^, ^10^ ^]^ They can secrete a large panel of bioactive molecules to modulate immune imbalance and promote regenerative pathways in refractory RA. The therapeutic effect of intravenously delivered ADSCs, on the other hand, is greatly challenged by short‐term retention and inadequate biodistribution in the target site.^[^ ^11^, ^12^, ^13^, ^14^ ^]^ As an alternative, various hydrogel vehicles that can provide immune isolation for stem cells are put forward.^[^ ^7^, ^15^, ^16^, ^17^, ^18^, ^19^, ^20^, ^21^ ^]^ Although with much progress, the existing hydrogel vehicles still face conundrums of suboptimal encapsulation and poor injectability, restricting their clinical translation.^[^ ^22^, ^23^, ^24^, ^25^, ^26^, ^27^ ^]^ Additionally, most reported hydrogels lack immunoregulatory capability on ADSCs and cannot guarantee their sufficient therapeutic effects.^[^ ^28^, ^29^, ^30^ ^]^ Therefore, developing new injectable hydrogel vehicles capable of sustaining the survival and promoting the function of implanted ADSCs is still eagerly anticipated.

**Figure 1:**
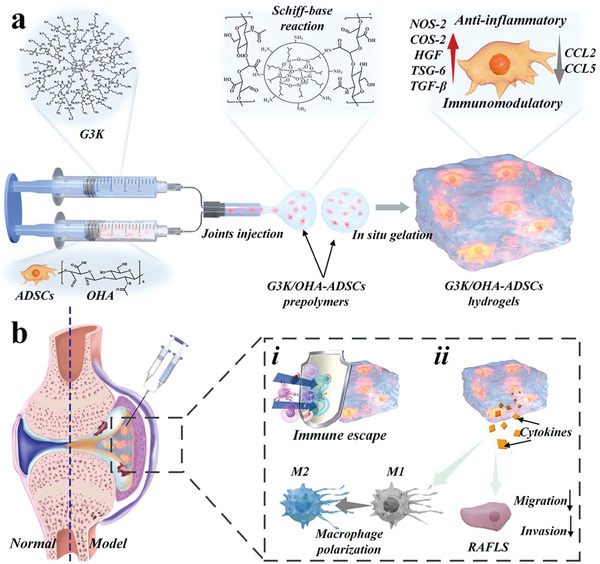
Schematic demonstration of the ADSCs‐laden bioinspired hydrogel for RA treatment through in situ injection in inflamed joints. a) Reaction scheme to show the crosslinking of G3K and OHA via the Schiff‐base reaction and the immunomodulatory functions of G3K/OHA‐ADSCs hydrogel. b) Schematic diagram of G3K/OHA‐ADSCs hydrogel to regulate the cell communication network toward anti‐inflammation in inflamed joints of RA.

Here, inspired by the chemical components of natural extracellular matrix (ECM), we developed a novel injectable hydrogel vehicle formed by dendritic polylysine and hyaluronic acid (HA) to retain ADSCs’ bioactivity for RA treatment, as shown in **Figure** **1**. ECM is a hierarchically hydrogel‐like network of filamentous substances, including polysaccharides and polypeptides.^[^ ^31^, ^32^ ^]^ It affords structural and mechanical support for cell growth and phenotype regulation.^[^ ^33^, ^34^ ^]^ To give cells an ECM‐like microenvironment, peptides, like dendritic polylysine, or polysaccharides, like chitosan, alginate, and HA, were used to make injectable hydrogel vehicles.^[^ ^35^, ^36^, ^37^, ^38^, ^39^ ^]^ However, because each polysaccharide and polypeptide only has one function, current ECM‐mimicking hydrogels struggle to mimic the fibrillar architecture and biological function of natural ECM. Additionally, the injectable and self‐healing behaviors of composite hydrogels remained controversial and have yet to be explored.

In this paper, we conceived that the biomimetic construction of injectable hydrogels with polysaccharides and polypeptides structures, simultaneously, would provide a distinctive strategy for ADSCs encapsulation and RA treatment. To implement this concept, generation three polylysine dendrimer (G3K) and oxidized hyaluronic acid (OHA) were employed as the central components of the ECM‐inspired hydrogel. Through the reversible Schiff base crosslinks between aldehydes on the HA chain and amines on the periphery of G3K, the resultant G3K/OHA hydrogel was endowed with injectable and self‐healing capacity. Besides, the hydrogel scaffolds exhibited superior mechanical properties, providing structural support and a microenvironment for cell growth. Based on these, we have demonstrated that the ADSCs‐laden ECM‐inspired hydrogel could reverse the dominant macrophage phenotype from M1 to M2 and prevent the migration of RA fibroblast‐like synoviocytes (FLS). In addition, the proposed ADSCs hydrogel could considerably attenuate RA symptoms in vivo, including joint swelling, bone destruction, and cartilage damage. These features make the bioinspired hydrogel with ADSCs encapsulation valuable in treating RA and various autoimmune diseases.

## 2 Results and Discussion

OHA was synthesized by a ring‐opening reaction of HA using sodium periodate (NaIO_4_) as oxidant. By regulating the molar ratios of HA to periodate (2:1, 1:1, 1:2), three oxidation degrees of OHA were obtained (59.46%, 87.84%, and 37.5%), respectively. The oxidation degree was validated by a hydroxylamine hydrochloride titration method (Table S1, Supporting Information). OHA with the most aldehyde functional groups (87.84%) was selected as a gel precursor. ^1^H‐NMR spectra further revealed the characteristic chemical shift at 6.75 ppm, confirming the formation of aldehyde groups (Figure S1, Supporting Information). The third generation of dendritic polylysine (G3K) was synthesized in a solid phase, as described previously.^[^ ^40^, ^41^, ^42^ ^]^ The successful fabrication of G3K was confirmed by ^1^H‐NMR (Figure S2, Supporting Information). Then, through Schiff base reactions, the sol–gel transition occurred relatively quickly between G3K and OHA without needing other initiators or irradiation, as visualized by tilting the test tubes (Figure S3a, Supporting Information). To rationalize the physical performances to meet the clinical demands of cell engraftment, hydrogels with three different concentrations (10%, 20%, and 30% w/v) were formed and used for subsequent studies.

Proper gelation time is of great importance for injectable hydrogels. A slow crosslinking rate usually causes the leak and delocalization of loaded cells. Whereas, a fast gelation process limited the syringe ability of hydrogels. By decreasing the precursor concentration from 30% to 10%, the gelation time of G3K/OHA hydrogels could be flexibly adjusted from 5 to 40 s (Figure S3b, Supporting Information). Higher prepolymer concentrations led to faster gelation. Appropriate swelling ratio and biodegradability other prerequisites for hydrogel vehicles used in vivo. After being incubated with phosphate buffer solution (PBS, pH 7.4) for 2 d, 30% G3K/OHA hydrogel reached a relatively low equilibrium swelling ratio of 170% (Figure S4b, Supporting Information). We hypothesized that the rigid framework dendrimer limited gel expansion, which could prevent gel disintergration after in vivo application. In addition, the G3K/OHA hydrogels were fully biodegradable and the degradation rate was highly correlated with the crosslinking density (Figure S4c, Supporting Information).

**Figure 2:**
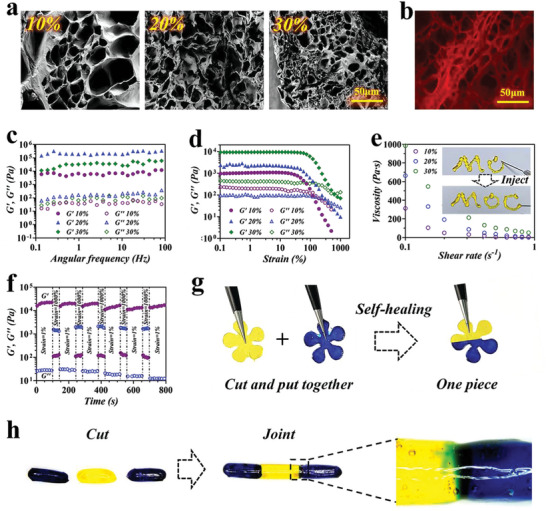
Characterization of G3K/OHA hydrogels. a) SEM and b) CLSM analysis of the hydrogel structure. *G*’ and *G*’’ of G3K/OHA hydrogels on c) frequency and d) strain sweeps. e) Change of steady shear viscosity as a function of shear rate for the hydrogel. The inset picture showed the shear‐thinning and injectability features of the hydrogel. f) Continuous step‐strain measurements were applied to the hydrogel in the step of 1% and 1000% oscillatory strain for cycles. g) Self‐healing process of G3K/OHA hydrogel with flower shapes. h) Blue and yellow stained cylinder‐shaped hydrogel were cut into three 5 mm length strips and recombined color by color. The joint position of two gels was observed under microscopy.

The microstructural change in hydrogels might determine their final mechanical properties. Scanning electron microscopy (SEM) and Confocal laser scanning microscope (CLSM) analysis revealed a homogeneous interconnective porous structure of G3K/OHA hydrogels (**Figure** **2**a,b and Figure S4a, Supporting Information). Then, the dynamic rheological tests, including frequency and strain sweeps, were conducted to evaluate the mechanical property of G3K/OHA hydrogels. As expected, hydrogels with different concentrations and a constant molar ratio of 1:1 between aldehydes and amines all showed a classical viscoelastic behavior with storage modulus (*G*’) greater than loss modulus (*G*’’) in the whole frequency ranges (Figure 2c). In the strain sweeps, 30% G3K/OHA hydrogels displayed relatively consistent *G*’ and *G*’’ values within 100%. Notably, gel disintegration occurred until the strain reached 1000%, suggesting the hydrogel could resist to relatively large deformation in tissue area with frequent activity, such as joints (Figure 2d). Compress/stretch experiments were further performed to assess the mechanical properties of hydrogel vehicles (Figure S5a,b and Movies S1 and S2, Supporting Information). In consistency with rheology tests, the elastic moduli of the hydrogels increased from 400 to 1100 kPa and the extensibility decreased from 105% to 25% by changing the gel concentration from 10% to 30% (Figure S5c, Supporting Information). The average energy loss of all hydrogels was less than 50% (Figure S5d, Supporting Information), suggesting their outstanding deformation resistance ability.

Shear‐thinning property is a essential requirement for injectable hydrogels. The G3K/OHA hydrogel showed a sharp decrease in viscosity with the increase in shear rate, indicating a dramatic gel–sol transition (Figure 2e). To further study the shear‐thinning behavior, the hydrogel precursors were prepared and transferred into a double‐barrel syringe with a 26‐gauge needle for injection. It was observed that the hydrogel network underwent destruction and was slowly squeezed out of the syringe (the inset picture of Figure 2e and Movie S3, Supporting Information). Interestingly, the hydrogel reformed into a gel state instantly after injection, suggesting the favorable injectability of G3K/OHA hydrogels.

The immediate self‐repair capacity of hydrogel was then investigated by cyclic oscillatory strain sweeps between 1% and 1000% at a continuous frequency of 10 Hz. As expected, a large strain at 1000% led to transient destruction of the hydrogel network. Notably, the microstructure was utterly rebuilt in less than 100 s as the *G*’ recovered rapidly to the normal value (Figure 2f). The G3K/OHA hydrogel demonstrated effective structure restoration over step changes in strain. To make the self‐healing property of the hydrogels more visualized, we cut off two pieces of flower‐shaped hydrogels with different colors and combined them, respectively (Figure 2g). It was noted that the two hydrogel pieces with different colors could reunite into one integral piece immediately, without any pressure applied. This excellent shear‐thinning and self‐healing property was ascribed to the dynamic equilibrium of Schiff base linkages.

To investigate the mechanical strength of the self‐healed gels, three cylinder‐shaped G3K/OHA hydrogels were cut and heterogeneously joined together. After 20 min of healing, the reunited hydrogel cylinder was stretched up to 500% strain (Figure S6 and Movie S4, Supporting Information). It was observed that the reforming hydrogel remained stable and intact under a large external force. Additionally, the connected boundaries between two gels became obscure over time under microscopy, intuitively demonstrating the self‐healing process of G3K/OHA hydrogels (Figure 2h).

As demonstrated above, G3K/OHA hydrogels showed superior mechanical properties, favorable injectability, self‐healing ability, proper swelling ratios, and biodegradation capability, all promising virtues of acting as stem cell vehicles. Thus, ADSCs were loaded into G3K/OHA hydrogel for RA treatment. First, flow cytometry was used to assess the ability of bioinspired G3K/OHA hydrogels to maintain cell stemness, as ADSCs are distinguished by the expression of specific cell surface markers CD105 and CD90, and the absence of CD34 and CD45.^[^ ^43^, ^44^ ^]^ The results showed that the expression of distinctive cell surface markers CD105 and CD90 was >95%, in parallel with CD34 and CD45 expression <2% (Figure S7, Supporting Information).

**Figure 3:**
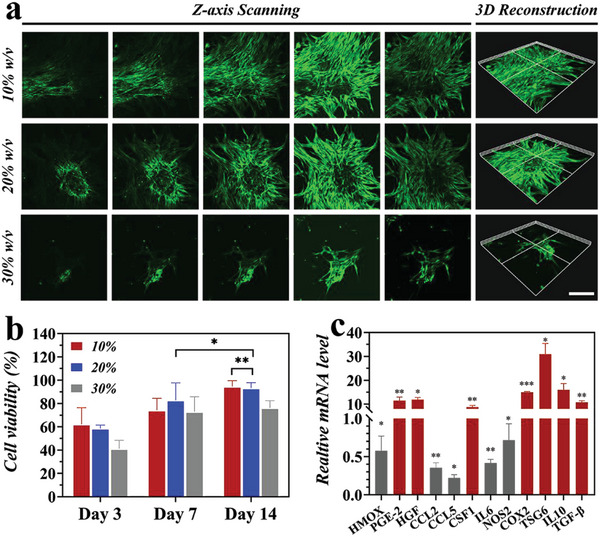
Hydrogel facilitated ADSCs cellular morphology, proliferation, and immunomodulatory function in vitro. a) CLSM images showed the ADSCs proliferation after 14 d of culture in G3K/OHA hydrogels. The scale bar is 200 µm. b) CCK‐8 assay of ADSCs encapsulated within the hydrogel for 14 d, cells cultured in tissue culture flask served as control. c) Gene expression of cytokines produced by ADSCs in the hydrogel, which was normalized to that of ADSCs on tissue culture. *n* = 3. Data were presented as mean ± Standard Deviation (SD). Statistical significance was calculated by one‐way analysis of variance (ANOVA) followed by post hoc tests, *0.01 < *P* < 0.05, **0.001 < *P* < 0.01, ***P < 0.001.

To validate the potential therapeutic utility of G3K/OHA hydrogels as stem cell vehicles, we investigated the cytocompatibility of encapsulated ADSCs by *Z*‐axis scanning and 3D reconfiguration analysis. When gel concentration is relatively low (10% and 20% w/v), the encapsulated ADSCs were distributed evenly, and all displayed a long shuttle‐shaped spread morphology after 14 d of coculture, confirming the formation of highly interconnected cellular networks (**Figure** **3**a). In contrast, oval cell aggregation could be observed in the hydrogels with high concentrations (30% w/v), and most ADSCs could not expand. The cell counting kit‐8 (CCK‐8) assay also revealed considerable cell proliferation from day 3 to day 14 for all hydrogel samples (Figure 3b). The remarkable biocompatibility of G3K/OHA hydrogels was mainly attributed to the ECM‐biomimetic chemical composition and the mild Schiff base crosslinking process, validating their promising values for long‐term ADSCs encapsulation. By collectively weighing the mechanical properties and cell‐friendly properties, 20% G3K/OHA hydrogels were selected in our subsequent studies.

A crucial prerequisite of hydrogel vehicles applied in stem cell engraftment is the positive immunoregulatory effects on stem cells. Therefore, a series of genes associated with rheumatoid arthritis were detected by reverse transcription‐polymerase chain reaction (RT‐PCR) quantification. Compared with control groups, ADSCs seeded within the G3K/OHA hydrogels showed a reduced gene expression related to inflammation, including HMOX, CCL5, CCL2, IL‐6, and iNOS. On the contrary, the mRNA expression of critical anti‐inflammatory mediators secreted by G3K/OHA‐ADSCs, like TSG‐6 and Interleukin (IL)‐10, was about tenfold greater than that of ADSCs seeded on cell plates (Figure 3c), suggesting a significantly improved immunomodulatory and anti‐inflammatory function of G3K/OHA‐ADSCs hydrogels. This anti‐inflammatory effect was caused by the cationic G3K molecules, which used ionic interactions to get rid of the inflammatory cytokines and cell debris that were negatively charged.^[^ ^40^, ^45^, ^46^, ^47^, ^48^ ^]^

**Figure 4:**
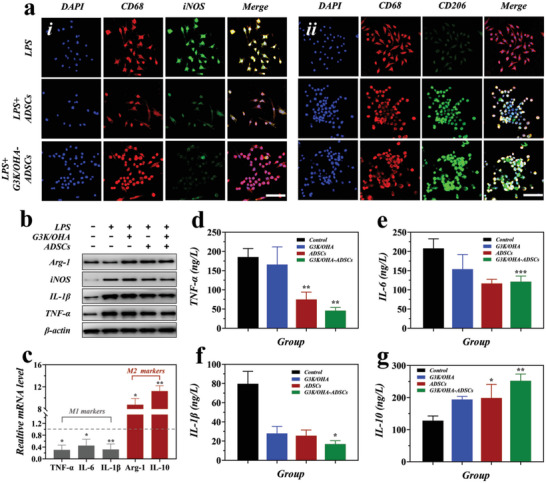
In vitro M1 to M2 polarization of BMDMs macrophages by G3K/OHA‐ADSCs hydrogels. a) Immunofluorescence staining of CD68 (the pan‐macrophage marker, red), iNOS (M1 marker, green), and CD206 (M2 marker, green), and nuclei (blue) on macrophages without or with the treatment of G3K/OHA‐ADSCs hydrogels. Scale bar = 50 µm. b) Protein expression of M1 (iNOS, IL‐1*β*, and TNF‐*α*) and M2 (Arg‐1) macrophage markers in BMDMs under various conditions, as evaluated by Western blot analysis. c) The mRNA levels of M1 macrophage markers (IL‐1*β*, IL‐6, and TNF‐*α*), and M2 macrophage markers (Arg‐1 and IL‐10) in activated macrophages without and with the treatment of G3K/OHA‐ADSCs hydrogels, as evaluated by qRT‐PCR analysis. d–g) Supernatant cytokines were detected in BDMDs treated by G3K/OHA, ADSCs, and G3K/OHA‐ADSCs. *n* = 3. Data were presented as mean ± SD. Statistical significance was calculated by one‐way ANOVA followed by post hoc tests, *0.01 < *P* < 0.05, **0.001 < *P* < 0.01, ***P < 0.001.

In RA joints, macrophages predominantly of the M1 phenotype could secrete various inflammatory cytokines and facilitate RA progression. Shifting macrophage polarization from M1 to M2 phenotype, determined by iNOS (M1 marker) and CD206 (M2 marker), respectively, becomes a promising strategy for RA treatment. Next, using rat bone‐marrow‐derived macrophages (BMDMs), we examined whether the G3K/OHA‐ADSCs could promote M2 polarization. The Purity of BMDMs was identified by expressing particular cell surface markers, CD11b and F4/80, using flow cytometry analysis (Figure S8, Supporting Information). By immunofluorescent labeling of the specific cell surface markers, BMDMs treated by the extracts of ADSCs and G3K/OHA‐ADSCs showed an increased signal of CD206 and a considerably downregulated level of iNOS, indicating a successful M1 to M2‐type switch (**Figure** **4**a). Western blot analysis results were in high accordance with that of the confocal assay. Reduced expression levels of proinflammatory mediators (iNOS, IL‐1*β*, and tumor necrosis factor (TNF)‐*α*) were observed after G3K/OHA‐ADSCs treatment (Figure 4b). Notably, G3K/OHA hydrogel, ADSCs, and G3K/OHA‐ADSCs all markedly enhanced the expression of M2 marker Arg‐1 on BMDMs.

To further confirm the macrophage reprogramming ability of G3K/OHA‐ADSCs, a series of genes associated with proinflammatory and anti‐inflammatory responses, including TNF‐*α*, IL‐6, IL‐1*β*, Arg‐1, and IL‐10, were detected by RT‐PCR. In comparison with the control group, macrophages cocultured with G3K/OHA‐ADSCs extracts showed a dramatic decrease in mRNA levels of TNF‐*α*, IL‐6, and IL‐1*β*, while upregulated the expression of Arg‐1 and IL‐10 (Figure 4c). Consistently, supernatant concentrations of TNF‐*α*, IL‐6, IL1*β*, and IL‐10 were detected by enzyme‐linked immunosorbent assay. It was seen that G3K/OHA‐ADSCs inhibited the production of proinflammatory cytokines and raised the level of anti‐inflammatory IL‐10 in BDMDs. All these results collectively demonstrate the anti‐inflammatory activity of G3K/OHA‐ADSCs by promoting macrophage differentiation to the anti‐inflammatory M2 subtype.

**Figure 5:**
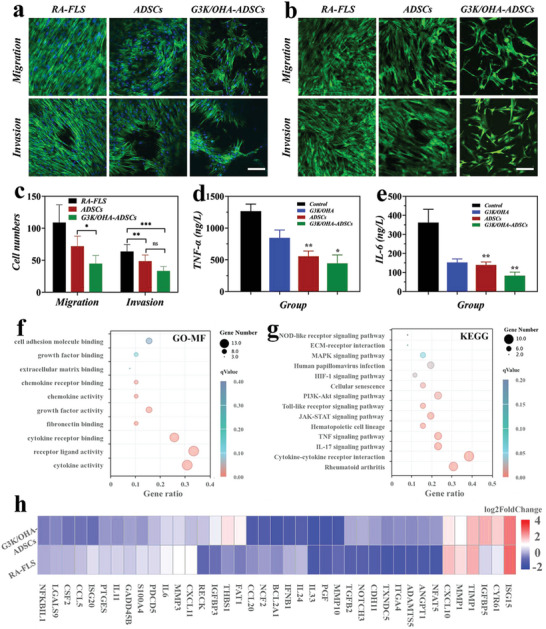
G3K/OHA‐ADSCs inhibited RAFLS migration, invasion, and inflammatory responses in vitro. a) Actin/diamidino phenylindole (DAPI) and b) Calcein acetoxymethyl (AM) staining of RAFLS in migration and invasion assays. The scale bar is 200 µm. c) Quantification of RAFLS cells migrated and invaded through Trans well. d,e) Supernatant proinflammatory cytokines were detected in RAFLS treated by G3K/OHA, ADSCs, and G3K/OHA‐ADSCs. f) GO and g) KEGG pathway analyses of the target genes of the top ten significantly expressed miRNAs in the ADSCs group compared with the NC group. The GO terms and KEGG pathway terms enriched in the predicted target genes of the miRNAs were analyzed using Database for Annotation Visualization and Integrated Discovery (DAVID) Bioinformatics. MF, molecular functions. h) The expression of marker genes in ADSCs and negative control (NC) groups. *n* = 3. Data were presented as mean ± SD. Statistical significance was calculated by one‐way ANOVA followed by post hoc tests, *0.01 < *P* < 0.05, **0.001 < *P* < 0.01, ***P < 0.001.

By migrating, invading, and releasing proinflammatory mediators and matrix metalloproteinases (MMPs), RA fibroblast‐like synoviocytes (RAFLS) contribute to the destruction of collagen and cartilage in RA joints. Here, to investigate whether G3K/OHA‐ADSCs hydrogels could inhibit the migration and invasion of RAFLS, transwell experiments were first performed by incubating RAFLS with the culture supernatant from ADSCs and G3K/OHA‐ADSCs. The results showed that both ADSCs and G3K/OHA‐ADSCs could stop RAFLS from migrating and invading. However, G3K/OHA‐ADSCs were more effective at stopping RAFLS (**Figure** **5**a–c). Moreover, the production of proinflammatory cytokines (TNF‐*α* and IL‐6) by RAFLS was significantly inhibited after treatment by G3K/OHA‐ADSCs, indicating a crucial role of G3K/OHA‐ADSCs in regulating FLS function and RA progression (Figures 5d,e).

To find out how G3K/OHA‐ADSCs stop FLS from migrating and invading, RNA sequencing (RNA‐seq) experiments were done on FLS that incubated with G3K/OHA‐ADSCs extracts (ADSCs group) and Matrigel (NC group). In comparison with the NC group, the ADSCs group showed a differential upregulation of 3678 genes and downregulated 3279 genes (*p* < 0.001) (Figure S9, Supporting Information). GO enrichment analysis showed that the effects of G3K/OHA‐ADSCs on FLS were related to the top ten differentially expressed miRNAs, such as “cytokine activity” and “receptor ligand activity” in molecular functions, “leukocyte migration” and “extracellular matrix organization” in biological processes, and “endoplasmic reticulum lumen” and “proteinaceous extracellular matrix” in cellular components. (Figure 5f and Figure S10, Supporting Information). Signaling pathway enrichment was detected through KEGG analysis. There were 14 highly enriched pathways linked to FLS function in the development of RA. These included rheumatoid arthritis, cytokine‐cytokine receptor interaction, IL‐17 signaling pathways, TNF signaling pathways, janus kinase‐signal transducer and activator of transcription (JAK‐STAT) signaling pathways, nucleotide oligomerization domain (NOD)‐like receptor signaling, and ECM receptor interaction (Figure 5g). Heatmap analysis revealed the top 39 markers genes with significantly different expression in highly enriched pathways (Figure 5h).

To investigate whether hydrogel enhances the retention of ADSCs in tissue, luciferase‐labeled ADSCs were injected into the joints of Sprague‐Dawley rats to recapitulate the survival of ADSCs in a functional immune system. Near‐infrared fluorescence imaging was applied to evaluate the fluorescent intensity of the recipient animals at 0, 1, 3, and 7 d (Figure S11, Supporting Information). ADSCs‐laden hydrogels led to a longer in vivo retention time and a higher intensity of ADSCs compared to the bare ADSCs group at the end of 7 d. The shorter in vivo residence time of bare ADSCs may be due to the fact that they broke down faster when exposed to proteases. The results proved that the formation of G3K/OHA‐ADSCs hydrogel promoted cell viability and survival, leading to the increment of immunomodulatory function and therapeutic effects in vivo.

**Figure 6:**
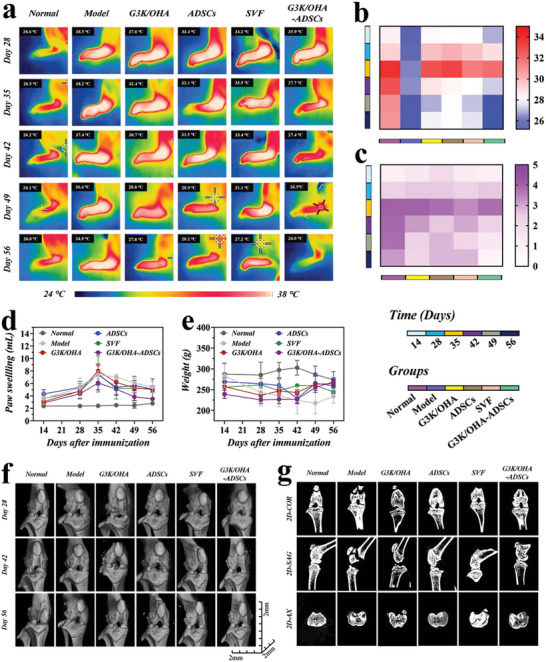
In vivo assessments of pathological features of CIA rat models after intra‐articular injection of various materials. a) Thermographic images of left hind paws and corresponding quantification of b) paw temperatures, c) clinical scores, d) paw swelling, and e) body weight at various time points after treatment. f) Representative 3D reconstructed micro‐CT images of the knee joints and corresponding 2D images in the COR, SAG, and AX planes. *n* = 5, biologically independent samples. Data were presented as mean ± SD.

The capability of G3K/OHA‐ADSCs hydrogels to facilitate cell proliferation, promote immunomodulation, modulate macrophage polarization, and inhibit RAFLS migration and invasion suggests their great translational potential. To assess the therapeutic effects of G3K/OHA‐ADSCs in vivo, we established a collagen‐induced arthritis (CIA) rat model with similar pathological presentations resembling human RA. Stromal vascular fraction (SVF) with microfragments (30–70 µm) of connective adipose tissue containing ADSCs served as the positive control because it worked as a natural‐derived cell scaffold, giving encapsulated ADSCs nutrients and mechanical support. All rats have already developed arthritis 28 d after immunization, with typical features of severe erythema and striking hyperthermia in joints observed by thermography (Figure S12, Supporting Information). After administering G3K/OHA hydrogel, ADSCs, SVF, and G3K/OHA‐ADSCs following the onset of CIA at day 28, paw temperature associated with inflammation was relieved to vary degrees (**Figure** **6**a). Beyond day 56, the paw temperature of the CIA rats remained relatively high, and paw volume increased to ≈214% (Figure 6b,d). On the contrary, we observed a dramatic decrease in both paw temperature and swelling of rats treated with G3K/OHA, ADSCs, and SVF. In the G3K/OHA‐ADSCs group, the treatment produced a more positive effect than other groups, and the hind paws swelling decreased to a nearly normal level at day 56. Similarly, the animals treated by G3K/OHA‐ADSCs showed the greatest improvement in clinical scores of hind paws (Figure 6c). Additionally, CIA rats exhibited a significantly weight loss at day 56 compared to the normal group, while the body weight in G3K/OHA, ADSCs, and G3K/OHA‐ADSCs treated groups all showed normal fluctuations (Figure 6e).

The microcomputed tomography (micro‐CT) imaging was also executed to check the influence of different treatments on bone destruction in the CIA rat. Though 3D reconstructed micro‐CT images and 2D reconstructed images in the coronal (COR), sagittal (SAG), and axial (AX) planes, it was easy to see bone erosion in the ankle (Figure S13a, Supporting Information) and knee (Figure 6f,g) joints of G3K/OHA, ADSCs, and SVF groups. Notably, G3K/OHA‐ADSCs outperformed its counterparts. The CIA rats treated by G3K/OHA‐ADSCs showed considerable enhancement in bone repair and were almost identical to normal rats. Treatment with G3K/OHA, ADSCs, and SVF between led to a slight improvement in the bone mineral density (BMD) of ankle (Figure S13b, Supporting Information) and knee (Figure S13c, Supporting Information) compared to the model group. It was noteworthy that the BMD of the G3K/OHA‐ADSCs group was recovered to 1758 and 1969 mg cm^−3^, separately, comparable to that of the normal rat, indicating their remarkable efficiency in suppressing bone destruction.

**Figure 7:**
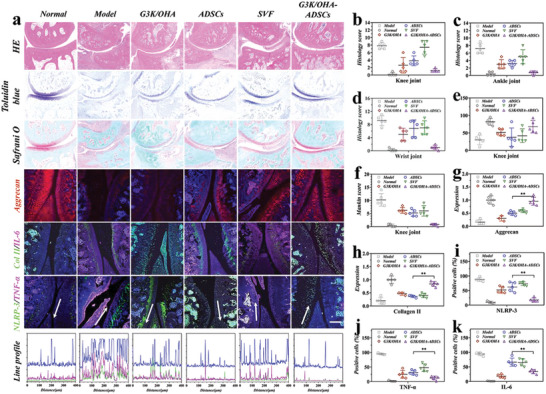
Evaluation of the therapeutic efficacy after the administration of G3K/OHA‐ADSCs. a) Tissue sections were stained with H&E, toluidine blue (TOL B), safranin O (SAF O), aggrecan, Collagen II (Col II), IL‐6, TNF‐*α*, and NLRP3. Line profile analysis confirmed the coexpression and colocalization of NLRP3 and TNF‐*α*. Histology scores of the b) knee, c) ankle, and d) wrist joints of CIA rats in different groups. e) Cartilage thickness of knee joints in different groups. f) Mankin's scores of knee joints in different groups for the quantification of cartilage integrity. Relative g) aggrecan content and h) collagen II in different groups after treatment. The percentage of i) NLRP3, j) TNF‐*α*, and k) IL‐6 ‐positive cells in synovial tissue of different groups. Scale bar, 200 µm. *n* = 5, biologically independent samples. Data were presented as mean ± SD. Statistical significance was calculated by one‐way ANOVA followed by post hoc tests, *0.01 < *P* < 0.05, **0.001 < *P* < 0.01, ***P < 0.001.

On day 56 after immunization, the knee, ankle, and twist joints of rats were all collected for histological analysis. The H&E, Toluidine blue, and Safranin O staining revealed an intense neutrophil infiltration, glycosaminoglycans (GAGs) loss, and cartilage damage in the model group (**Figure** **7**a). In contrast, the G3K/OHA, ADSCs, SVF, and G3K/OHA‐ADSCs treated groups showed a considerable enhancement in histology results, in which the G3K/OHA‐ADSCs group displayed the best therapeutic outcomes, demonstrating that the synergistic therapeutic effect of ECM‐inspired hydrogel scaffolds combined with ADSCs. Sections of knee (Figure S14, Supporting Information and Figure 7e), ankle (Figure 7a), and twist joints (Figure S15, Supporting Information) stained with Toluidine blue and Safranin O showed that the G3K/OHA‐ADSCs group had more GAG deposition and much clearer and thicker cartilage boundaries than the other groups, similar to those of the healthy group. This suggests that G3K/OHA‐ADSCs are a powerful way to protect cartilage when treating RA. The damage to the cartilage was also measured using the corresponding histology (Figure 7b–d) and Mankins (Figure 7f) scores.

Type II collagen (Col II) and aggrecan, two typical markers expressed by healthy chondrocytes, were detected by immunofluorescent staining. Normal rat joints showed a strong and widespread intensity of aggrecan/Col II signals (Figure 7a and Figures S16 and S17, Supporting Information). In contrast, the production of aggrecan/Col II proteins in the model group was significantly decreased. We found that G3K/OHA, ADSCs, and SVF treatments all help aggrecan/Col II expression to some degree. (Figure 7g,h). As expected, G3K/OHA‐ADSCs remarkably increased the synthesis of proteoglycan and Col II, protecting chondrocytes from degeneration.

Proinflammatory factors NLRP3, TNF‐*α*, and IL‐6 also play key roles in RA progression. The production of NLRP3, TNF‐*α*, and IL‐6 in G3K/OHA‐ADSCs groups were significantly decreased compared to any of the single treatments, indicating the encapsulation of ADSCs in ECM‐inspired hydrogels acted as effective cell therapies for RA treatment (Figure 7a and Figures S17 and S18, Supporting Information). Line profile analysis confirmed the coexpression and colocalization of NLRP3 and TNF‐*α*. The NLRP3, TNF‐*α*, and IL‐6 expression levels were quantitatively characterized (Figure 7i–k), respectively. Even though ADSCs and hydrogels alone reduced the number of immune cells, lytic lesions were still seen. This suggests that stem cell engraftment or ECM composition alone may not be enough to alleviate RA progression.

The H&E analysis of the heart, liver, spleen, lung, and kidney was also performed to evaluate the in vivo biocompatibility of G3K/OHA‐ADSCs. Notably, no major organ lesions were observed in all treated groups, suggesting their superior biocompatibility, consistent with their minimal in vitro cytotoxicity (Figure S19, Supporting Information). Also, no hepatotoxicity and nephrotoxicity were detected (Figure S20, Supporting Information). The serum level of alkaline phosphatase, alanine aminotransferase, aspartate aminotransferase, creatinine, urea, and uric acid were all comparable to that of normal rats. The ECM‐inspired components of G3K/OHA‐ADSCs and the mild crosslinking process through the Schiff base reaction may explain their minimal toxicity.

## 3 Conclusion

In this work, we developed an ECM‐inspired injectable self‐healing hydrogel composed of peptide dendrimer and OHA with ADSCs encapsulation for RA treatment. The G3K/OHA hydrogel was endowed with shear‐thinning and self‐healing capabilities via the dynamic equilibrium of Schiff base linkages. The resultant hydrogel scaffolds showed superior mechanical properties due to the high symmetrical nanostructure of peptide dendrimer. Importantly, the ECM‐like components of the G3K/OHA hydrogel could not only promote cell growth but also improve the immune‐regulating functions of encapsulated ADSCs in vitro. Moreover, this G3K/OHA‐ADSCs hydrogel could reprogram the macrophage phenotype to an anti‐inflammatory type and inhibit the migration and invasion of RAFLS in vitro. We have also demonstrated that the G3K/OHA‐ADSCs hydrogel could considerably attenuate RA symptoms in vivo by using a CIA rat model. As a result, this research shed new light on the exploration of effective engineered stem cell therapy for RA treatment.

## Conflict of Interest

The authors declare no conflict of interest.

## Author Contributions

The author contribution is as follows: investigation, formal analysis, data curation, writing original draft (H.Z.); visualization, investigation (X.W.); validation (R.L.); conceptualization, methodology, supervision (Y.Z.); and conceptualization, methodology, supervision (L.S.).

## Supporting information

Supporting Information

Supplemental Movie 1

Supplemental Movie 2

Supplemental Movie 3

Supplemental Movie 4

## Data Availability

The data that support the findings of this study are available in the Supporting Information of this article.
