# Correction to “ECM‐Inspired Hydrogels with ADSCs Encapsulation for Rheumatoid Arthritis Treatment”

**DOI:** 10.1002/advs.202417661

**Published:** 2025-01-22

*Adv Sci*. 2023;*10*(9):e2206253

1. The fluorescent image of cell staining in **Figure** **5**a (RAFLS/Migration and G3K/OHA‐ADSCs/Invasion group) was wrongly used during the assembly of Figure 5. We tracked down the original data obtained on September 16, 2021, and have replaced Figure 5a (RAFLS/Migration and G3K/OHA‐ADSCs/Invasion group) with the correct image as follows:

**Figure 5:**
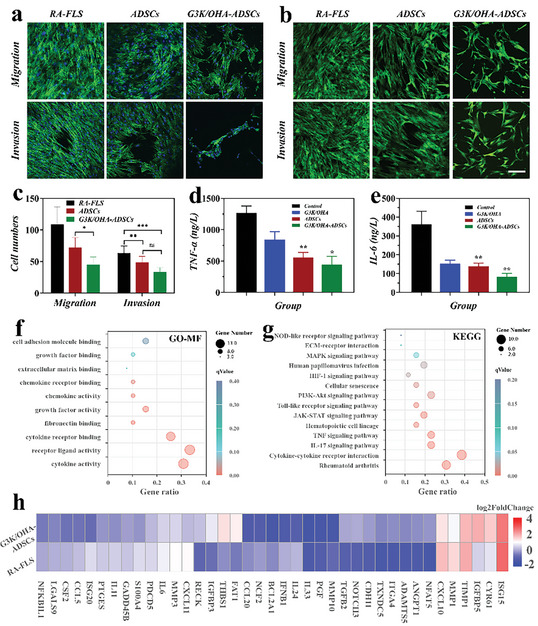
G3K/OHA‐ADSCs inhibited RAFLS migration, invasion, and inflammatory responses in vitro. a) Actin/DAPI and b) Calcein AM staining of RAFLS in migration and invasion assays. The scale bar is 200 µm. c) Quantification of RAFLS cells migrated and invaded through Trans well. d, e) Supernatant pro‐inflammatory cytokines were detected in RAFLS treated by G3K/OHA, ADSCs, and G3K/OHA‐ADSCs. f) GO and g) KEGG pathway analyses of the target genes of the top ten significantly expressed miRNAs in the ADSCs group compared with the NC group. The GO terms and KEGG pathway terms enriched in the predicted target genes of the miRNAs were analyzed using DAVID Bioinformatics. MF, molecular functions. h) The expression of marker genes in ADSCs and NC groups. *n* = 3. Data were presented as mean ± SD. Statistical significance was calculated by one‐way ANOVA followed by post hoc tests, ^*^0.01 < *p* < 0.05, ^**^0.001 < *p* < 0.01, ^***^ *p* < 0.001.

2. The Micro‐CT images in **Figure** **6**f (Model/Day 28 group) were wrongly used during the assembly of Figure 6. We tracked down the original data obtained on October 20, 2021, and have replaced Figure 6f (Model/Day 28 group) with the correct image as follows:

**Figure 6:**
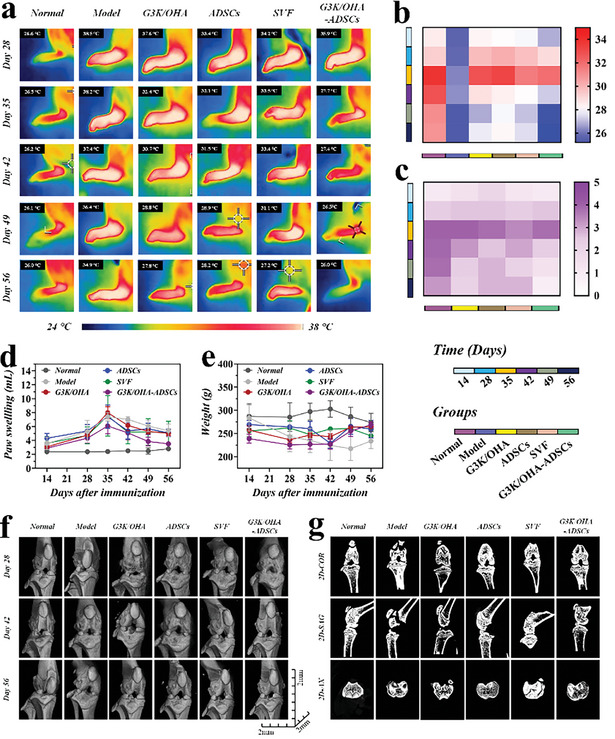
In vivo assessments of pathological features of CIA rat models after intra‐articular injection of various materials. a) Thermographic images of left hind paws and corresponding quantification of b) paw temperatures, c) clinical scores, d) paw swelling, and e) body weight at various time points after treatment. f) Representative 3D reconstructed micro‐CT images of the knee joints and corresponding 2D images in the COR, SAG, and AX planes. *n* = 5, biologically independent samples. Data were presented as mean ± SD.

These errors do not affect the results or conclusions of the paper.

We apologize for this error.

