# Supplementary material for: ECM‐Inspired Hydrogels with ADSCs Encapsulation for Rheumatoid Arthritis Treatment
Source: Adv Sci (Weinh). 2023 Jan 22;10(9):2206253. doi: 10.1002/advs.202206253 (PMC10037981; doi:10.1002/advs.202206253)
Supplement: Supplementary file 1 — Supporting Information [file ADVS-10-2206253-s005.pdf]

Supporting Information

ECM-inspired Hydrogels with ADSCs Encapsulation for Rheumatoid Arthritis Treatment

Haofang Zhu[a], *Xiangyi Wu*[a], *Rui Liu*[a], *Yuanjin Zhao*[a,b,]*, *Lingyun Sun*[a]*

Methods

Materials: (3-Aminopropyl)-triethoxysilane was commercially available from Aladdin Co., Ltd (Shanghai, China). H-Lys-OMe 2HCl, Boc-Lys(Boc)-OH, HOBT, HBTU, DIEA, and TFA were commercially available from Aladdin Co., Ltd (Shanghai, China). HA (Mw=100 kDa) was purchased from Bloomage Biotechnology Corporation Limited. CCK-8 assay kit, Calcein AM, and propidium iodide (PI) were commercially available from KeyGEN BioTECH Co., Ltd (Nanjing, China). Alexa Fluor® 750 NHS Ester (succinimidyl ester) (AF750-NHS, Invitrogen) was dissolved in DMSO to prepare a stock solution of 10 mg/mL. Phosphate buffered solution (PBS, pH = 7.4), Dulbecco's modified Eagle medium (DMEM), and fetal bovine serum (FBS) were purchased from United States Origin, Gibco. Mouse TNF-α ELISA Kit (Biolegend), Mouse IL-6 ELISA Kit (Biolegend), Mouse IL-1β ELISA Kit (Biolegend), Mouse IL-10 ELISA Kit (Biolegend), Rat TNF-α ELISA Kit (Biolegend), Rat IL-6 ELISA Kit (Biolegend), Rat IL-1β ELISA Kit (Biolegend), Rat IL-10 ELISA Kit (Biolegend), Human TNF-α ELISA Ready-SET-Go! (eBioscience), and Human IL-6 ELISA Ready-SET-Go! (eBioscience) were purchased accordingly. Immunization grade bovine type II collagen solution (2 mg/mL, Chondrex), complete

Freund's Adjust (4 mg/mL, Chondrex), and isoflurane (RWD Life Science, China) were purchased from companies.

Synthesis and characterization of G3K: Polyhedral oligomeric silsesquioxane (POSS) core-based generation 3 poly($_L$-lysine) dendrimers (POSS-G3K) were synthesized as described previously. ^{1}H NMR (Bruker, Germany, 400 MHz) spectra was used to confirm the synthesis of G3K.

Synthesis and characterization of OHA: HA aqueous solution (1% w/v, dissolved in 100 mL of distilled water) with different molar ratios to $NaIO_4$ (2:1, 1:1, 1:2) was stirred overnight in the dark at ambient temperature of 25 °C to yield OHA with appropriate degree of oxidation. 1 mL diethylene glycol was then added to quench any unreacted $NaIO_4$. The solution was stirred for 1 hour following by dialyzing against distilled water for 3 days. The resulting OHA dry product was obtained by lyophilization. The oxidization degree of OHA was quantified by measuring the number of aldehyde groups in polysaccharide chains according to a standard protocol slightly modified. OHA (20 mg) was dissolved in hydroxylamine hydrochloride solution (5 mL, 0.25 M) of pH 3. The mixture was stirred gently overnight at ambient temperature of 25 °C. Then the oxidization degree was measured by the titration of the hydrochloric acid (HCl), which is generated by the reaction between aldehyde groups with hydroxylamine hydrochloride. The titration was conducted with standardized sodium hydroxide aqueous solution (NaOH, 0.1 M) till the end point has been reached at pH 3 as recorded on a digital pH meter. The oxidization degree of

OHA that was defined by comparing the titer volume values of NaOH (0.1 M) with that obtained from standard curve, produced by the titer volume value of NaOH (0.1 M) against the amount of 2,4-dihydroxybenzaldehyde. The oxidation degrees of OHA obtained by three different molar ratios (2:1, 1:1, 1:2) were determined as 59.46%, 87.84%, and 37.5%, respectively. The structure of OHA was further confirmed by ^{1}H NMR (Bruker, Germany, 400 MHz) spectra.

Fabrication of G3K/OHA hydrogels: OHA with an oxidation degree of 87.84% was selected to crosslink with G3K. At room temperature, OHA (0.1, 0.2, and 0.3 g) and G3K (0.1, 0.2, and 0.3 g) were separately dissolved in 1 mL phosphate buffered saline (PBS, pH 7.4) to form 10%, 20%, and 30% w/v prepolymer solutions. Then OHA and G3K prepolymer solutions with same concentration were mixed evenly at volume ratio of 1:1 to form 10%, 20%, and 30% w/v G3K/OHA hydrogels, respectively, by a dual barrel syringe.

Morphology Characterization: For scanning electron microscopy (SEM) observation, the lyophilized hydrogels were placed on double-sided electric tape and sputter-coated with gold before examining. Confocal laser scanning microscope (CLSM) images were obtained by using a Leica Microsystems (Leica TCS SP5, Germany), in which G3K was fluorescently labelled with AF750 for microscopic observation.

Rheological Analysis of G3K/OHA Hydrogel: Rheological test was carried out on an AR2000ex rheometer (RS-5, TA Instruments) with a cone and plate geometry (diameter 25 mm, gap size 300 μm) at 25 °C. The storage modulus G′, loss modulus

G″ and viscosity were both analyzed for these studies. (1) G′and G″of the pregelled G3K/OHA hydrogel discs were tested under a 1% strain, and the angular frequency was set from 0.1 to 100 Hz. (2) G′ and G″ of G3K/OHA hydrogel versus strain were tested a fixed frequency of 10 Hz and an increasing strain from 0.01% to 10000%. (3) The alternate step strain sweep of G3K/OHA hydrogel was measured at 10 Hz angular frequency, and amplitude oscillatory strains were changed from $\gamma = 1\%$ to 1000% with 120 s for every strain interval. (4) The shear-thinning behavior was investigated using a steady shear flow measurements which determine the viscosity changes in wide shear rate range (0.1–100 s^{-1}).

Swelling and degradation ability of hydrogels: The swelling ratio measurements of free-dried hydrogel were carried out gravimetrically. The known weights of free-dried hydrogels were immersed in the PBS at 37°C. At specific intervals of time, the swollen samples were taken out and weighted. All experiments were carried out in triplicate. The swelling ratio was calculated using the following equation: Swelling ratio (%) = (Ws − Wd)/Wd × 100%, where Ws and Wd were the weights of hydrogels at the swelling state and the dry state. To investigate the tunable degradation of the G3K/OHA hydrogel in wet environment, the prepared hydrogel was lyophilized and initial dry weight was measured (W_1). The degradation behavior of hydrogel was studied by PBS 7.4 incubation in a shaker incubator (37 °C, 50 rpm) and PBS was exchanged every 12 hours for 14 days. Samples were removed from the PBS and the remained weight was measured after lyophilization at 1st, 7th, and 14th day post

incubation (W_d). The weight remaining (%) was calculated using the following equation: $W_d/W_1 \times 100$.

Injectability and Self-Healing Performance of Hydrogels: A piece of G3K/OHA hydrogel with 20 min pregelation time was added into 1 mL syringe with needle (26-gauge) and injected onto a glass slide to form designated letters. Two pieces of heart-shaped G3K/OHA hydrogel with 20 min pregelation time stained into yellow and blue color were cut into equal two parts, respectively. After 20 min at 25 °C, the total four pieces of alternate colors were combined into two integral blended heart-shaped pieces. Self-healing was confirmed by the capacity of the healed flower-shaped hydrogel to hold its structure when suspended under gravity. Three cylinder-shaped G3K/OHA hydrogels were operated as the steps described above. After 20 min of healing, the reunited hydrogel cylinder was stretched. The above process was recorded by Cannon 60D as photographs and movie as well as microscopy images.

Tensile and cyclic compression tests: Tensile and cyclic compression tests were performed using a universal testing machine (UTM, Instron 4466 tester). 100 μL pre-gel solution was placed into polydimethylsiloxane (PDMS) rectangular (20 × 5 × 1 mm) molds for tensile tests, the resultant hydrogels were extended with a loading rate of 1 mm min^{-1} until structural failure. Cylindrical (diameter 8 mm, gap size 2 mm) molds were used for compressive tests, the hydrogels were cyclically loading and unloading for 5 cycles at a strain rate of 1 mm min^{-1}. Energy loss was determined by calculating the area between the loading and unloading stress/strain curves. (n = 5)

Cell cultures: For ADSC isolation, subcutaneous adipose tissues from patients were digested with 250 U/mL collagenase type II for 1 h at 37 °C and centrifuged (300 g for 10 min) using routine laboratory practices. Prior to surgery, all patients have signed a written informed consent. All studies were performed according to recognized ethical guidelines (2020-072-01) issued by Ethics Committee of Drum Tower Hospital (Nanjing, China). The SVF was collected and cells filtered successively through a 100 μm, 70 μm and 40 μm porous membrane (Cell Strainer, BD-Biosciences, Le-Pont-de-Claix, France). ADSCs were seeded at the initial density of 4000 cell/cm2 in αMEM supplemented with 10% FBS and 1% penicillin/streptomycin (PS). To isolate rat BMDMs, bone marrow was collected from the femurs of 5-week-old male SD rat, as described previously.[49] First, hind limbs of animals were collected and muscle tissue was removed to expose the femur bones. Bones were then flushed with PBS using syringes to isolate bone marrow cells. After centrifugation, collected bone marrow cells were differentiated to macrophages for 4 days in differentiation medium composed of high-glucose DMEM supplemented with 15% (v/v) FBS and 1% (v/v) penicillin/streptomycin. Primary RA-FLS were purchased from ATCC and grown in a DMEM/F12 medium containing 10% heat-inactivated FBS, in a humidified incubator at 37 °C under 5% CO_2.

Flow cytometric assay: Flow cytometric assays were performed to test cell surface markers expression. For ADSC identification, after two weeks of encapsulation in the G3K/OHA hydrogels, released ADSCs were collected and incubated with the

antibodies of CD105, CD90, CD45, and CD34 (all from eBioScience, CA, United States). For BMDM identification, after one week of culture, BMDMs were collected and incubated with the antibodies of CD11b and F4/80 (all from eBioScience, CA, United States). The flow cytometric analysis was conducted with BD FACSCalibur flow cytometer (BD Biosciences). The data was analyzed with FlowJo version 10. Cells with CD105/CD90 positivity above 95% and CD45/CD34 positivity below 5% were considered to possess stemness. Cells with CD11b and F4/80 positivity above 95% were identified as macrophages.

Biocompatibility evaluation: ADSCs were calculated and suspended in 1 mL of G3K precursor solution at 1 × 10^6 cells per mL. The cell-laden G3K solution was added into OHA solution to generate ADSCs encapsulated G3K/OHA hydrogels (G3K/OHA-ADSCs). Then the G3K/OHA-ADSCs were incubated in DMEM/F-12 media under 5% CO_2 for 14 days. On day 14, G3K/OHA-ADSCs were washed with DPBS (Dulbecco's PBS) repeatedly to remove calcium and magnesium ions, then the G3K/OHA-ADSCs were stained with 10 μL Calcein AM and PI for 5 minutes. Then the G3K/OHA-ADSCs were washed by DPBS repeatedly to remove excess Calcein AM and PI followed by immersing in fresh DPBS solutions for CLSM analysis (Olympus, FV-3000, Japan). For CCK-8 assay, the G3K/OHA-ADSCs with different concentrations were submerged in DMEM/F12 media in a shaking incubator. After 24 hours of incubation, the supernatant was taken and co-cultured with ADSCs in a 96-well plate at a density of 1 × 10^5 cells per well for 72 hours. At 3-, 7-, and 14-days

post incubation, CCK-8 assay kit was used to quantitatively measure the cytotoxicity at each time point. A Bioreader (Biosys, Germany) was used to record the optical density (OD) value at 450 nm. The relative cell viability (%) was calculated by normalizing the OD value of cells incubated with microgel extracts to OD value of control cells cultured with pure DMEM/F-12 media at each time point.

Macrophage phenotype transition study: For immunofluorescence staining, BMDMs were seeded in a 24-well plate with coverslips and activated with LPS (10 μg/mL) for 48 h. The cells were then incubated with the supernatant of G3K/OHA-ADSCs for 48 h, followed by fixing with 4% paraformaldehyde. Then, the cells were incubated with primary antibodies against CD68 (1:100), iNOS (1:50) or CD206 (1:50) at 4 ℃ overnight and then incubated with respective fluorescent-labelled secondary antibodies for 30 min. The nuclei were stained with DAPI and the cells were mounted. The samples were observed with CLSM. For RT-PCR quantification, BMDMs were seeded onto 6-well plates at density of 2×10^5 cells per well and activated by LPS (10 μg/mL) for 48 h. Then, the supernatant of G3K/OHA-ADSCs were added. After 48 h, the cells were harvested and total RNA was extracted using TRIzol reagent. The levels of mRNAs (including IL-1β, IL-6, TNF-α, Arg-1 and IL-10) were determined by RTPCR. Primer sequences were designed by soft of primer 5.0 (Table S2). For Western blot analysis, cell lysates in RIPA Lysis buffer were boiled and centrifuged, and the supernatant was then separated by 10% SDS PAGE. The proteins on the PAGE were transferred to a PVDF membrane and probed with the antibodies against

Arg-1 (1:1000), iNOS (1:2000), IL-1β (1:2000), TNF-α (1:2000), and β-actin (1:2000), followed by the HRP-conjugated Goat Anti-Rabbit IgG second antibody (1:5000). The hybridized bands were then visualized with ELC assay by FluorChem R (ProteinSimple, America).

In vivo biodistribution and retention of ADSCs: The biodistribution of the luciferase-labeled ADSCs was monitored by NIRF imaging using an *in vivo* imaging scanner (IVIS Lumina LT, Perkin Elmer) at different time points during 7 days.

Animal model induction and treatment: All in vivo experiments were performed according to the Ethics Committee of Drum Tower Hospital and the approval number issued by the Laboratory Animal Welfare Ethics Committee of Drum Tower Hospital was 2021AE01008. The collagen-induced arthritis (CIA) rat model was established by tail intravenous injection of emulsion fabricated by a homogenizer with a small blade (diameter of 5 mm) to emulsify the IFA (4 mg/mL) with the collagen solution (2 mg/mL). On day 0, the rats were administrated with 200 μL emulsion. G3K/OHA, ADSCs, SVF, and G3K/OHA-ADSCs were injected to hindpaw joints once per week. Rats were monitored daily for arthritis progression. Hindpaws swelling were measured with a plethysmometer. After the mice were sacrificed, their hindlimbs were immediately collected and stored in paraformaldehyde (4%) at room temperature. The hindlimbs were assessed by micro-CT system (Sky- Scan, Bruker, Belgium). Scanning parameters were set at a voltage of 49 kV, current of 179 μA, and exposure time of 475 ms. The BMD of knee subchondral bone and anklebone were also

measured from reconstructed 3D CT images using the software provided by Bruker Company. In addition, the collected limbs were used for histological analysis using haemotoxylin and eosin (H&E), toluidine blue, safranin-O staining, and immunohistochemistry staining. The major organs were sectioned and stained by H&E. Staining was visualized using a digital microscope (Olympus, CX31, Japan). The criteria used to evaluate the H&E staining images of the different joints was: 0 = normal synovium; 1 = hypertrophy and cell invasion present in the synovial membrane; 2 = pannus and cartilage erosions present; 3 = erosions of cartilages and subchondral bone; and 4 = dysfunction and rigidity of the whole joint. All indicators of the scores were repeated twice, and the average scores were obtained. Blood samples from the mice were taken from the eye socket at day 70. The samples were centrifuged at 3000 rpm for 10 min after standing 2 h. The supernatant was collected and recentrifuged at 3000 rpm for 10 min again to obtain cell-free serum. The levels of alkaline phosphatase (ALP), alanine aminotransferase (ALT), aspartate aminotransferase (AST), creatinine, uric acid, and urea were measured by Automated Chemistry Analyzer (FAITH-1000, Nanjing Laura electronics co., LTD).*Statistical analysis:* All data were expressed as mean standard deviation (SD) values for at least five independent experiments. One-way analysis of variance (ANOVA) was followed by post hoc tests using SPSS 22.0 software to determine the significant difference between two groups. Values of *($p < 0.05$) were considered statistically significant.

Statistical Analysis: All the results were presented as the means ± standard deviation (SD). Statistical analysis was carried out using one-way ANOVA followed by Tukey's post-hoc test to determine the degree of significance by the software of Origin 2022. Statistical significance was defined as $^{*}p < 0.05$, $^{**}p < 0.01$, $^{***}p < 0.001$.

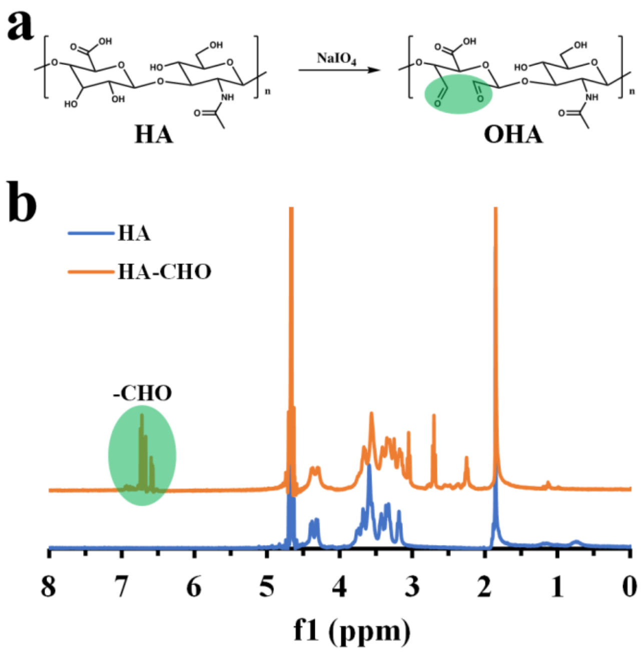

Fig. S1. Synthesis route of OHA and ^{1}H-NMR of HA and OHA. The characteristic chemical shift at 6.75 ppm confirmed the formation of aldehyde groups.

molar ratio [HA/$NaIO_4$]	Theoretical oxidization (%)	Obtained degree of oxidization (%)
1：0.5	50	37.5
1：1	100	87.84
1：2	100	59.46

Tab. S1. Determination of oxidization degree of OHA.

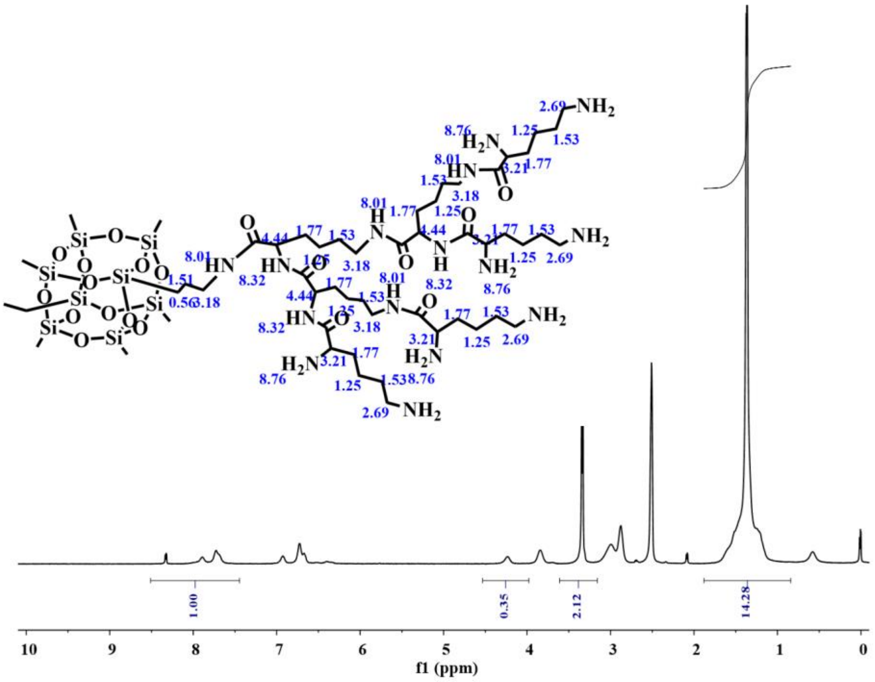

Fig. S2. ^{1}H-NMR of G3K.

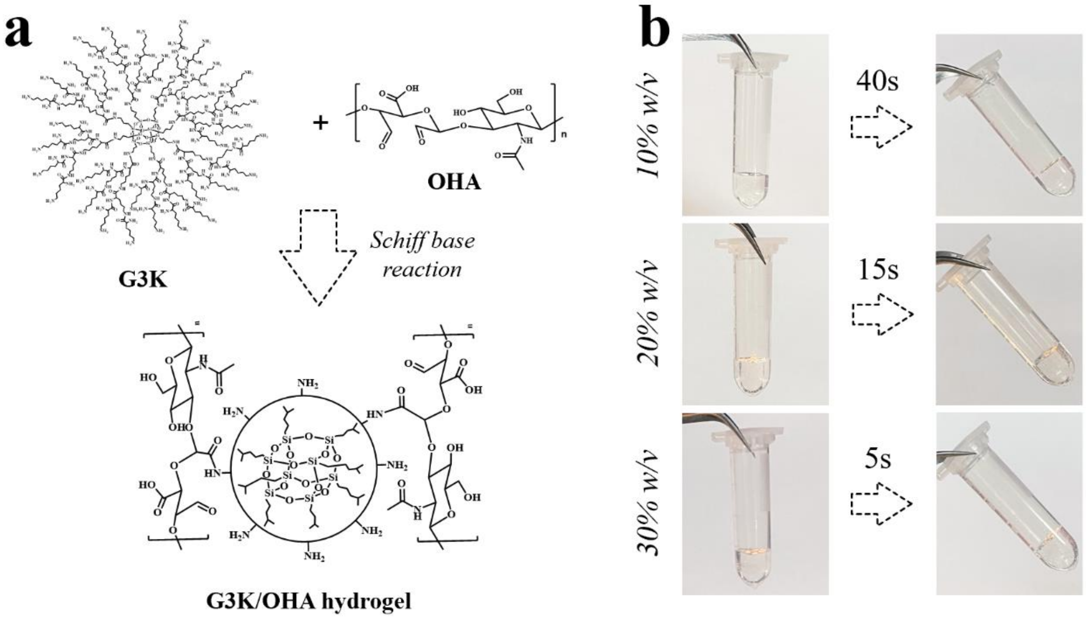

Fig. S3. a) Crosslinking mechanism of G3K/OHA hydrogels. b) Tilt tests of the gelation process of G3K/OHA hydrogels. The gelation time varied from 5s to 40s when the gel concentration increased from 10% to 30% w/v.

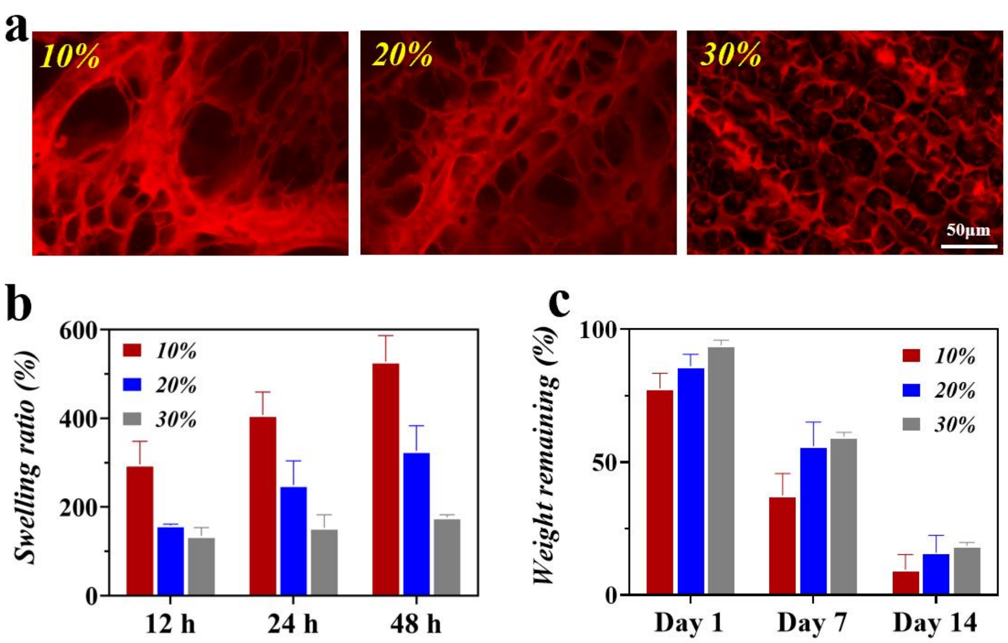

Fig. S4. The a) CLSM images, b) swelling ratios, and c) degradation ability of G3K/OHA hydrogels with different concentrations.

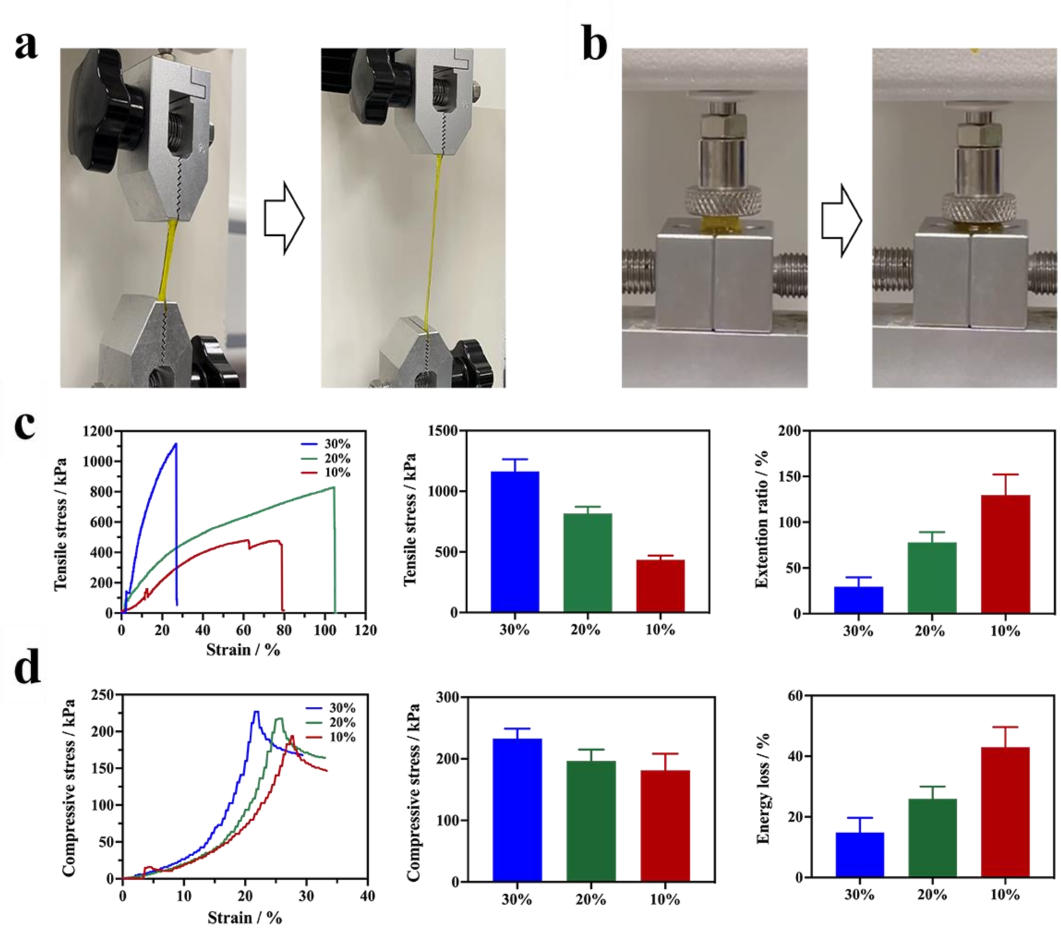

Fig. S5. a) Tensile and b) compression tests of G3K/OHA hydrogels with different concentrations. c) Representative tensile strain-stress curves, ultimate tensile strength, and elongation ratio at break of G3K/OHA hydrogels with different concentrations. d) Representative compression strain-stress curves, compressive modulus, and energy loss of G3K/OHA hydrogels with different concentrations.

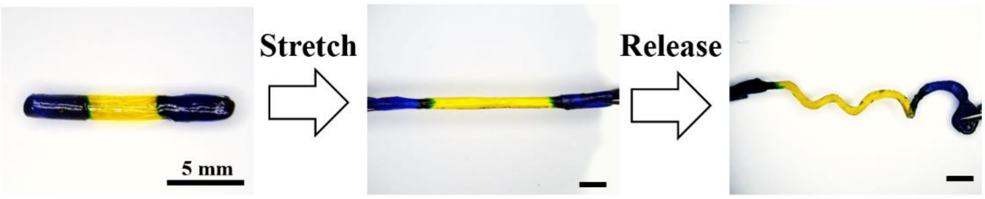

Fig. S6. The stretching process of the self-healed G3K/OHA hydrogels. After 20 min of healing, the reunited hydrogel cylinder was stretched up to 500% strain.

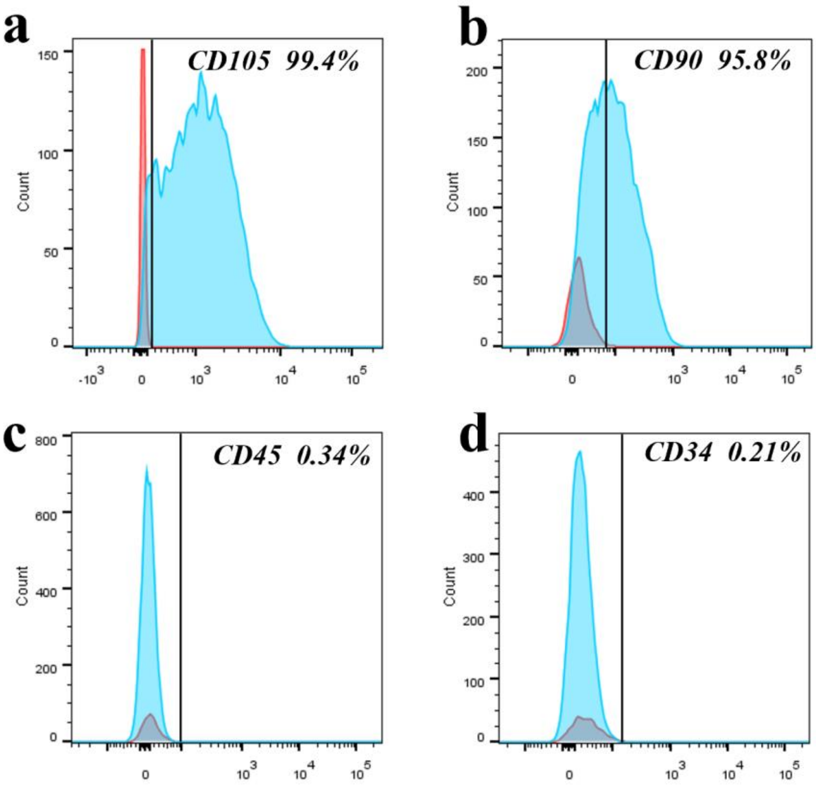

Fig. S7. Cell surface markers of ADSCs were assessed by flowcytometric analysis. Blue peaks were referred to as ADSCs stained with antibodies. Red peaks were referred to as ADSCs without staining.

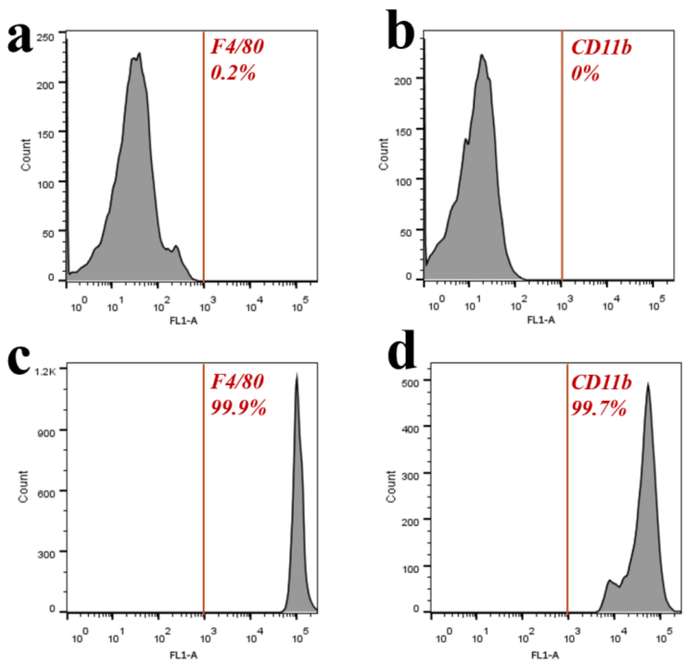

Figure S8. The purity of BMDMs was identified by flow cytometry and staining with anti-F4/80 antibody (FITC) and anti-CD11b antibody (APC). The representative scatter diagrams of each group are shown: the control group (A and B), the anti-F4/80 antibody group (C), and the anti-CD11b antibody group (D).

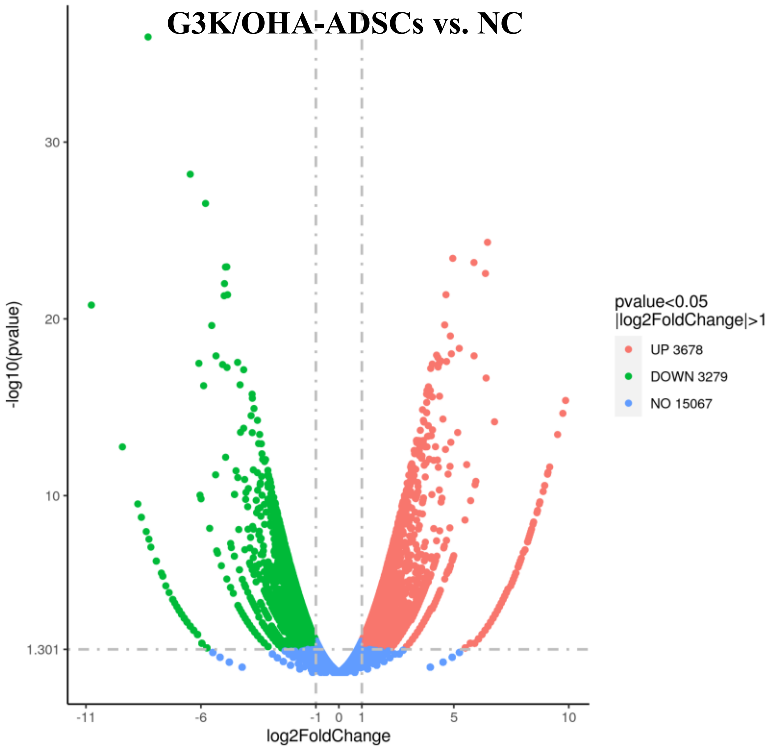

Figure S9. Differential gene statistics. The abscissa was Log2 Fold Change value, the ordinate was -Log10 (*p value*), and the dashed blue line represented the threshold line of the differential gene screening criteria.

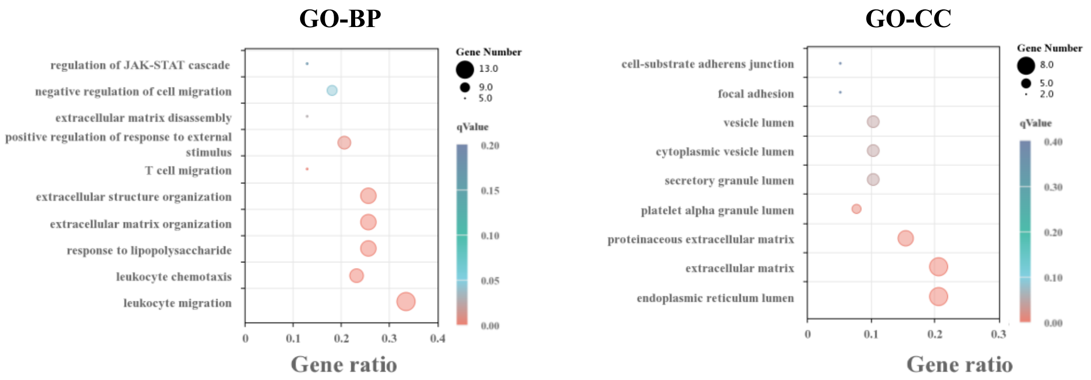

Figure S10. GO analysis of top ten significantly expressed genes in ADSCs group compared with NC group. BP, biological processes; CC, cellular components.

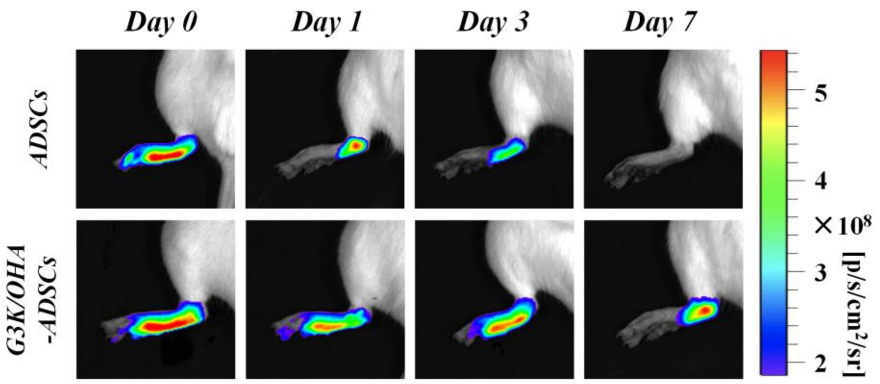

Fig. S11. Hydrogels promoted the retention of ADSCs *in vivo*. Representative luminescent signal images of ADSCs injected into SD rats' joints with or without hydrogel encapsulation at 0, 1, 3, and 7 days.

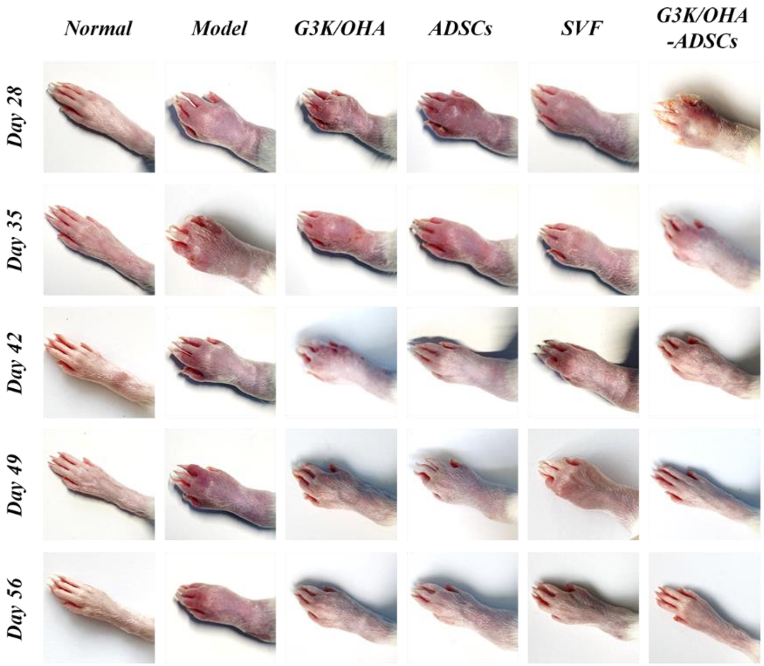

Fig. S12. Photos hindpaws in normal and G3K/OHA-ADSCs hydrogels treated RA model groups at day 28, 35, 42, and 56 after immunization.

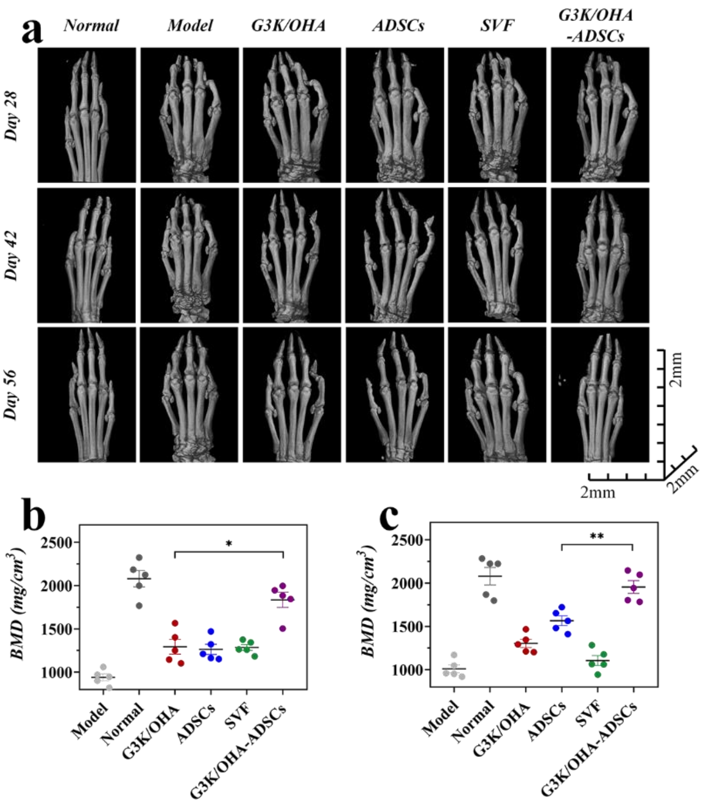

Fig. S13. a) Representative 3D reconstructed micro-CT images of the ankle joints and BMD of b) ankle joints and c) knee joints.

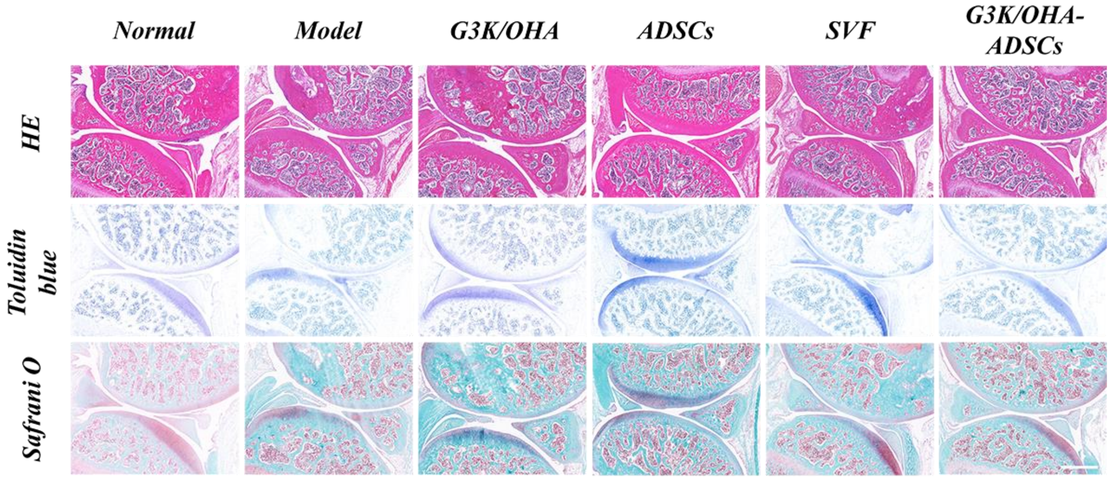

Fig. S14. Toluidine blue and safranin O staining of the knee joints.

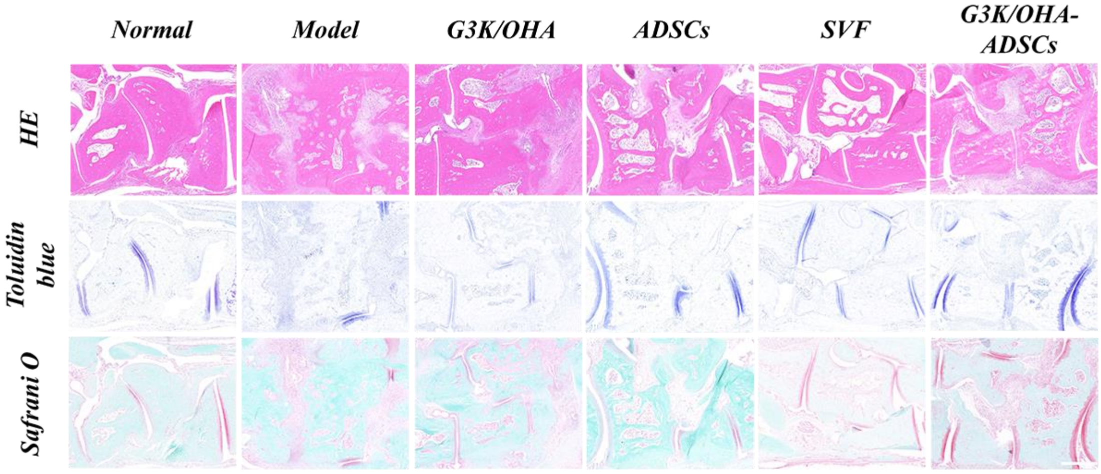

Fig. S15. Toluidine blue and safranin O staining of the twist joints.

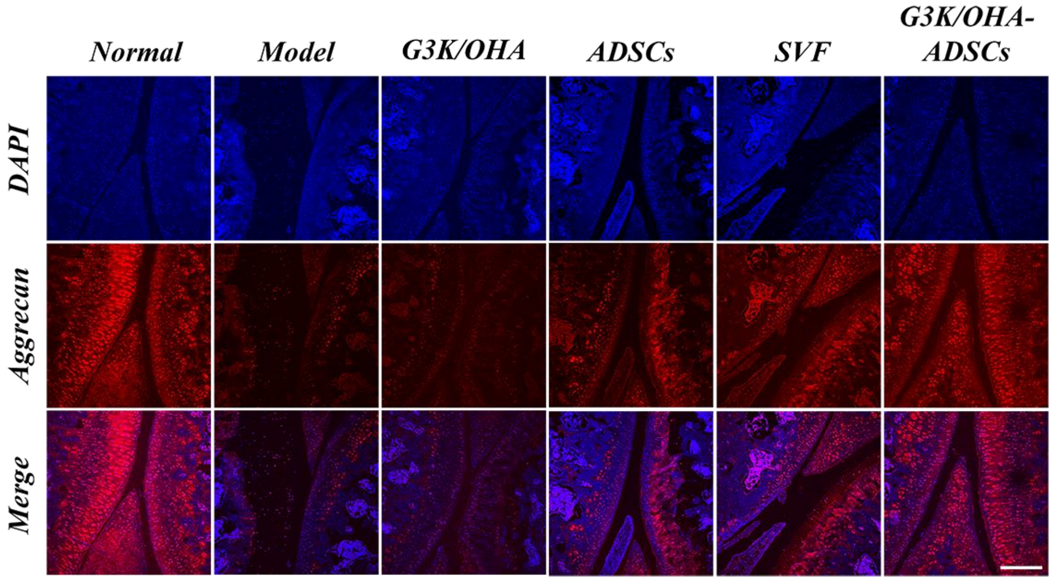

Fig. S16. Knee sections were stained with aggrecan and DAPI.

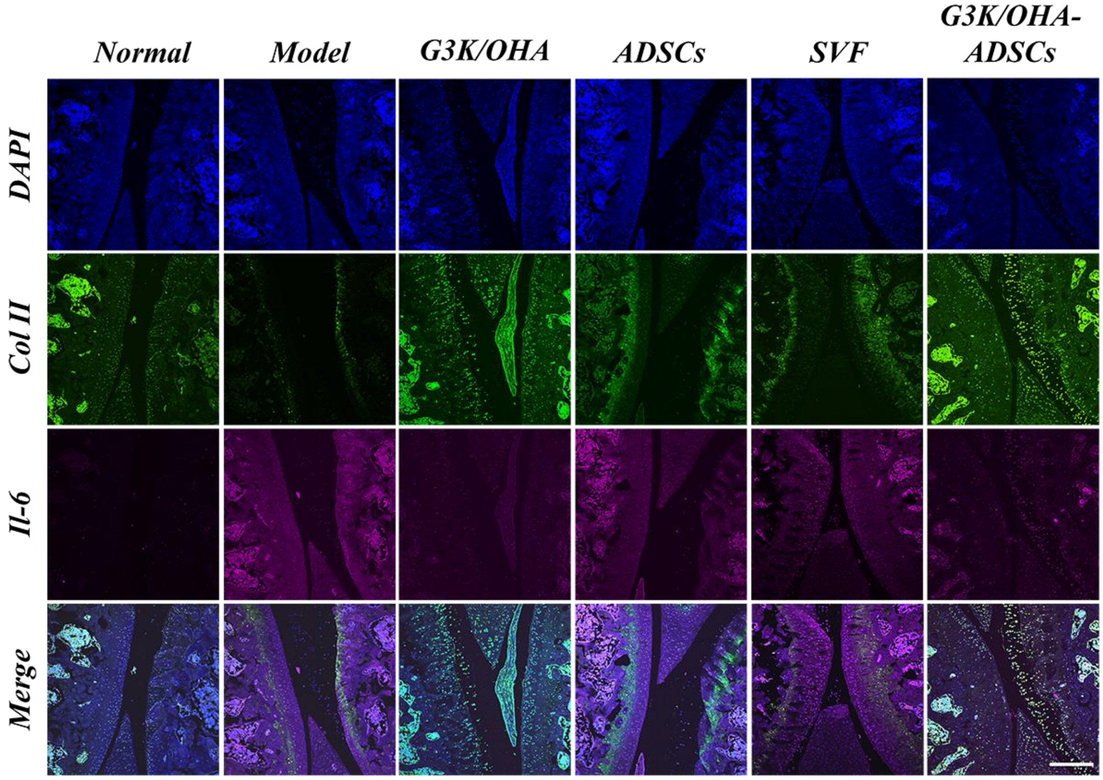

Fig. S17. Knee sections were stained with Collagen II (Col II), IL-6 and DAPI.

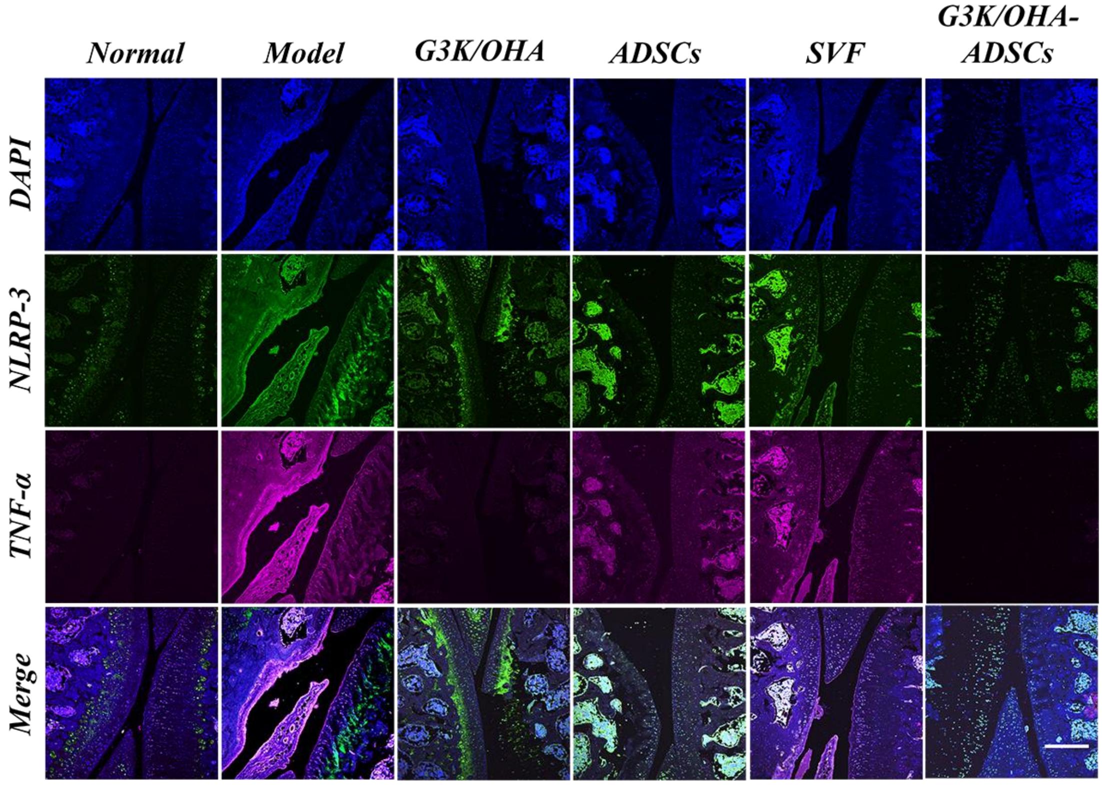

Fig. S18. Knee sections were stained with TNF-α, NLRP3, and DAPI.

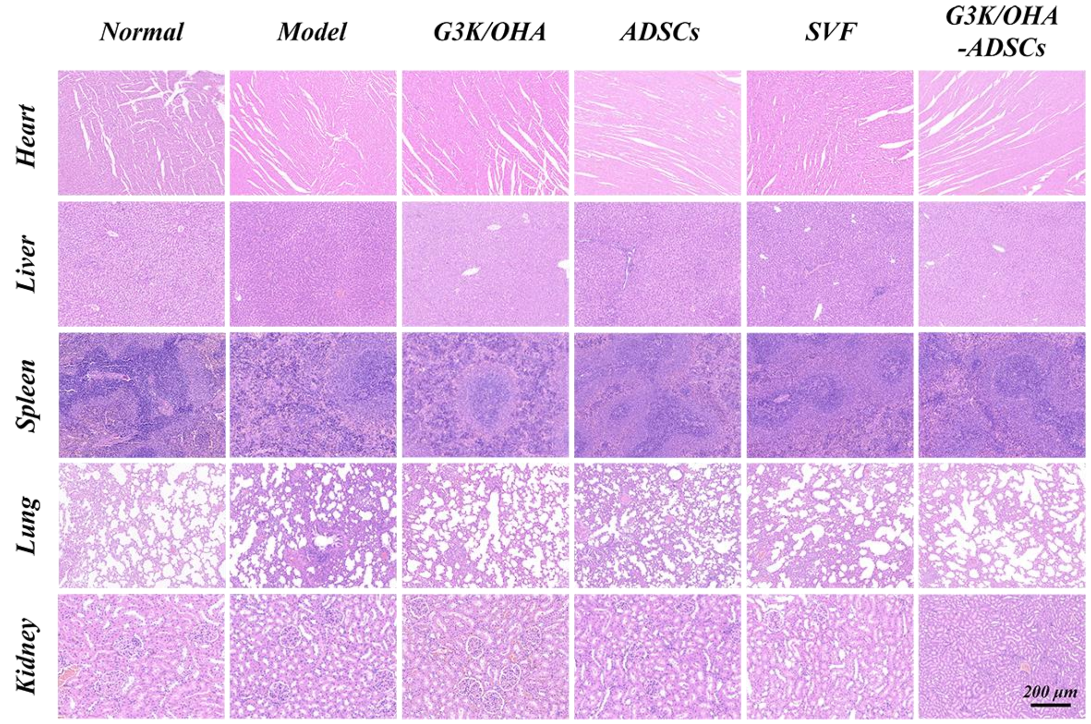

Fig. S19. H&E staining of heart, liver, spleen lung and kidney of rats.

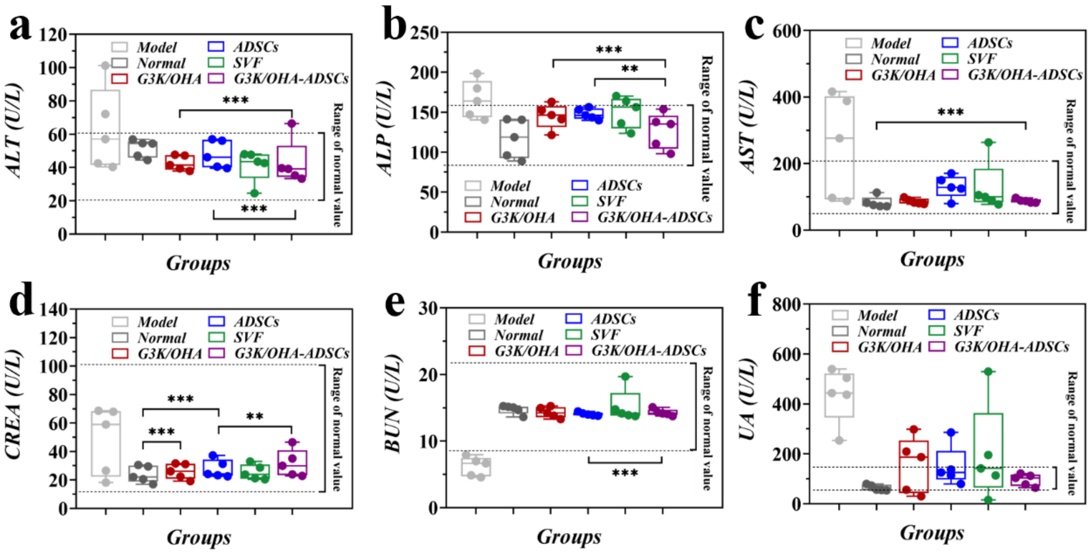

Fig. S20. ALP, ALT, AST, creatinine, urea, and uric acid analysis of CIA rats at day 56 (treatment for 28 d). Dash lines give the windows of normal ranges of each item.

Table S2 Primer sequences used for real-time PCR.

Gene	Forward Primer (5′ to 3′)	Reverse Primer (5′ to 3′)
IL-1β	ATGAAGGGCTGCTTCCAAAC	TCTCCACAGCCACAATGAGT
IL-6	GGAGCCCACCAAGAACGATA	ACCAGCATCAGTCCCAAGAA
TNF-α	CTCATGCACCACCATCAAGG	ACCTGACCACTCTCCCTTTG
Arg-1	TGGCTTGCGAGACGTAGAC	GCTCAGGTGAATCGGCCTTT
IL-10	CTGGACAACATACTGCTAACCG	GGGCATCACTTCTACCAGGTAA
GAPDH	GGGTCCCAGCTTAGGTTCAT	CCAATACGGCCAAATCCGTT
